# Microfluidics-Driven Dripping Technique for Fabricating Polymer Microspheres Doped with AgInS_2_/ZnS Quantum Dots

**DOI:** 10.1021/acsomega.4c07270

**Published:** 2024-09-02

**Authors:** Kamilla Kurassova, Nikita Filatov, Sofia Karamysheva, Anton Bukatin, Anton Starovoytov, Tigran Vartanyan, Frank Vollmer, Nikita A. Toropov

**Affiliations:** † International Research and Education Centre for Physics of Nanostructures, ITMO University, St. Petersburg 197101, Russia; ‡ Alferov Saint Petersburg National Research Academic University of the Russian Academy of Sciences, 8/3A Khlopina Street, St. Petersburg 194021, Russia; § Institute for Analytical Instrumentation of the Russian Academy of Sciences, 31-33A Ivana Chernykh Street, St. Petersburg 198095, Russia; ∥ Department of Physics and Astronomy, University of Exeter, Exeter EX4 4QD, U.K.; ⊥ Optoelectronics Research Centre, University of Southampton, Southampton SO17 1BJ, U.K.

## Abstract

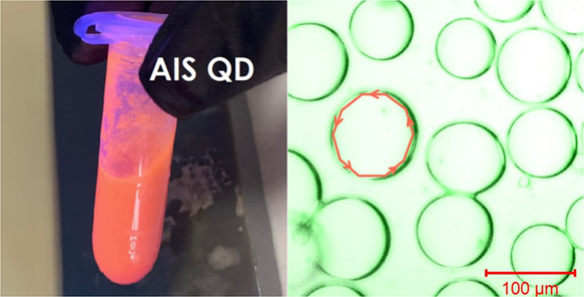

Fluorescent microspheres are at the forefront of biosensing technologies. They can be used for a wide range of biomedical applications. They consist of organic dyes and polymers, which are relatively immune to photobleaching and other environmental factors. However, recently developed AgInS_2_/ZnS quantum dots are a water-soluble, low-toxicity class of semiconductor nanocrystals with enhanced stability as fluorescent materials. Here, we propose a simple way for making microspheres: a microfluidic dripping technique for acrylamide polymer spheres doped with quantum dots. Analyses of their spectra show that the emission of quantum dots, dispersed in water, is saturated with an increasing pump intensity, while quantum dots embedded into polymer microspheres exhibit a more sustained emission. Moreover, our study unveils a remarkable reduction in the luminescence lifetime of quantum dots embedded in microspheres: the mean value of the decay time for quantum dots in solutions was 91 and 3.5 ns for similar quantum dots incorporated into polymer microspheres.

## Introduction

Fluorescent microspheres are of great importance in modern biosensing technologies.^1^ They can be used as essential tools for a wide range of biomedical applications including blood flow determination and tracing, in vivo imaging, calibration of imaging and flow cytometry instruments, absorbents, affinity bioseparators, and drug and enzyme carriers.^2^ Conventionally, they consist of organic dyes and polymers. The latter ones make dyes relatively immune to photobleaching and other environmental factors. However, semiconductor nanocrystals, namely, quantum dots (QDs), have proven themselves as more stable fluorescent materials than their organic counterparts.^3^ Recently, a new class of water-soluble low-toxicity quantum dots was developed, namely, AgInS_2_.^4,5^ Beside their photostability, QDs can be highlighted as their physical, chemical, and optical properties are tunable at the stage of their synthesis, sometimes via merely their size variation. These advantages allowed QDs to pave the way for numerous discoveries and experimental demonstrations, such as weak and strong coupling regimes in plasmon–exciton systems.^6^ The latter one relates to a regime of coupling when the rate of energy exchange between two subsystems is higher than the rate of energy dissipation in the coupled system. This led to the observation of Rabi splitting in the spectrum of a single quantum dot coupled to a single gold nanoparticle.^7^ On the other hand, the weak coupling regime manifests itself as a modification of luminescent spectra, in particular, as a metal-enhanced fluorescence caused by the Purcell effect.^6,8^ Meanwhile, active control of spontaneous emission from atoms, molecules, and QDs is highly desirable in modern quantum optics and nanophotonics.^9,10^

Thus, simple embedding of QDs into polymer microspheres may reveal interesting optical phenomena since the Purcell effect has already become of particular interest in such microspheres supporting whispering-gallery modes (WGMs).^9−11^ Such microspheres can be considered optical cavities which have unsurpassed values of their *Q*-factors.^12^ To date, there are a number of examples of WGM cavities doped with different fluorescent media, which also demonstrate lasing; they found impressive applications, e.g., in rapidly emerging areas of intracellular biosensing.^13−16^ Emissive WGM microspheres significantly broaden the opportunities for the advancement of such biosensors since the narrow resonance line width can be used for the analysis of biological tissues by a set of characteristics.^14,17^

In this article, we describe a method of making fluorescent and biodegradable^18,19^ microspheres doped with water-soluble AgInS_2_/ZnS QDs. This method exploits dripping microfluidics and allows microspheres to form with practically any required diameters. We analyzed the fluorescence of QDs incorporated in polymer microspheres and dissolved in water. The difference in their emission saturations was observed. We additionally investigated their fluorescence lifetime, which was drastically, 19-fold, reduced for QDs in microspheres.

## Materials and Methods

### AgInS_2_ Quantum Dot Synthesis

Quantum dot synthesis was performed according to the procedure described in ref (5). Briefly, the following components were sequentially added to water (96 mL) under magnetic stirring: 1.0 mL of aqueous AgNO_3_ (0.1 M), 2 mL of an aqueous thioglycolic acid (TGA) solution (1.0 M), and 0.2 mL of aqueous NH_4_OH (5.0 M). The resulting turbid light-yellow suspension becomes transparent after adding 0.45 mL of aqueous NH_4_OH and colorless after adding 0.7 mL of an aqueous InCl_3_ (1.0 M) solution in HNO_3_ (0.2 M). Next, 1.0 mL of an aqueous 1.0 M Na_2_S solution was added while stirring. The mixture at this stage had a pH equal to 8–9. The resulting solution was heated in a water bath at 90–95 °C for 30 min.

Subsequently, AgInS_2_ cores were encapsulated with a zinc sulfide (ZnS) shell (we will refer to such a structure as AIS) through the decomposition of the Zn^II^-TGA complex, enhancing the stability of their optical properties.^5^ For this purpose, 1.0 mL of an aqueous TGA solution (1.0 M) was added to 1.0 mL of an aqueous Zn(CH_3_COO)_2_ solution (1.0 M) in 0.01 M HNO_3_ under vigorous stirring. Then, the mixture was additionally heated for 30 min to grow the shell. At the end, the synthesized QDs were collected, evaporated, and precipitated by 2-propanol addition (1:2), followed by centrifugation at 4500 rpm for 5 min. The precipitate was separated and characterized (see the figures below).

### Microfluidic Synthesis of Acrylamide Microspheres

Microfluidic flow-focusing droplet generators were used to produce acrylamide microspheres with quantum dots. These devices were prepared from poly(dimethylsiloxane) (PDMS Sylgard 184, Dow Corning) by soft lithography.^20^ At first, a single-layer silicon mold was fabricated using the SU–8 2025 photoresist via contact optical lithography with a chromium mask. Then, the PDMS prepolymer and the curing agent were mixed in a ratio of 10:1 w/w, degassed, poured onto the mold, and cured at 65 °C for 2–4 h in an oven. After the curing step, the PDMS replicas with punctured inlet and outlet holes were bonded with cover glass slides by oxygen plasma or corona plasma treatment.

**Figure 1:**
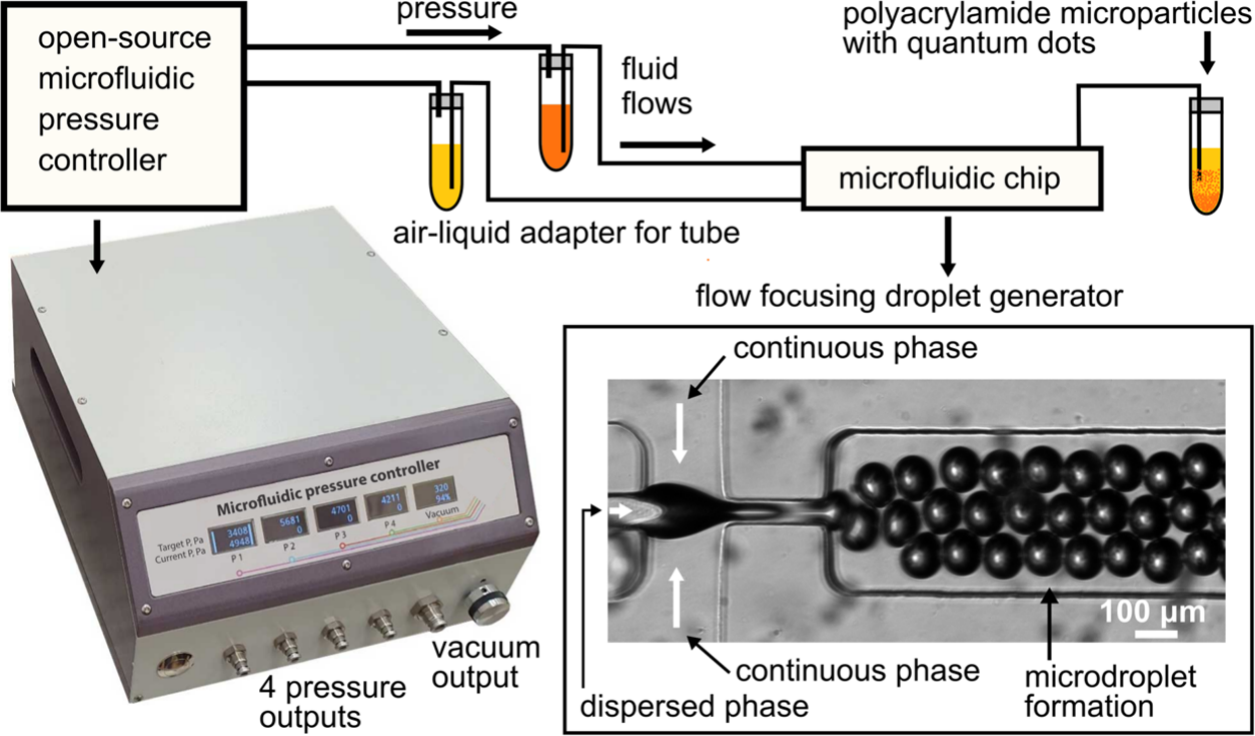
Circuit diagram of the experimental setup for acrylamide microspheres with quantum dot formation in a microfluidic device. The liquids were introduced into the device by the previously developed open-source microfluidic pressure controller.

To prepare acrylamide microparticles, mineral oil (Sigma-Aldrich, Merck) with the addition of a ∼3–3.5% ABIL EM 180 nonionic surfactant (Evonic Industries) was applied as a continuous phase. Also, tetramethylethylenediamine (TEMED), a polymerization catalyst, was added to the continuous phase at a concentration of 15 μL/mL. A water solution of the following components was used as the dispersed phase: (i) 635 μL of water (pure, deionized); (ii) 335 μL of 30% acrylamide/bis solution (29:1, Bio-Rad); and (iii) 30 μL of 10% water solution of an ammonium peroxydisulfate (Sigma-Aldrich, Merck) polymerization initiator.^21^ When adding quantum dots to the dispersed phase, 40 μL of a concentrated QD solution was used per 1 mL of the dispersed phase solution. The schematic view of the microfluidic experimental setup is presented in Figure 1. To introduce liquids into the chip, the open-source microfluidic pressure controller (MFPC) based on compact electro-pneumatic regulators was used.^22^ Briefly, under the required pressures, liquid reagents from laboratory tubes inserted into air–liquid adapters were introduced to the microfluidic chip through flexible Tygon tubing. Our previous studies showed that when using the pressure controller, the coefficient of variation (CV) of the “water-in-oil” droplet diameters was CV ≤ 4.3%. However, this parameter can be affected by the composition of the dispersed phase, microchannel design, and flow rates of the phases.^22,23^ Based on the size distribution histogram of the microspheres (see Figure 3d), the CV was calculated to be 19.78%, indicating a relatively large diversity in droplet sizes. Generally, droplets are considered monodisperse when the CV is less than 10%, meaning that their sizes are sufficiently uniform. With a CV of approximately 20%, the droplets in the sample are classified as polydisperse.

### Spectroscopy and Microscopy

**Figure 2:**
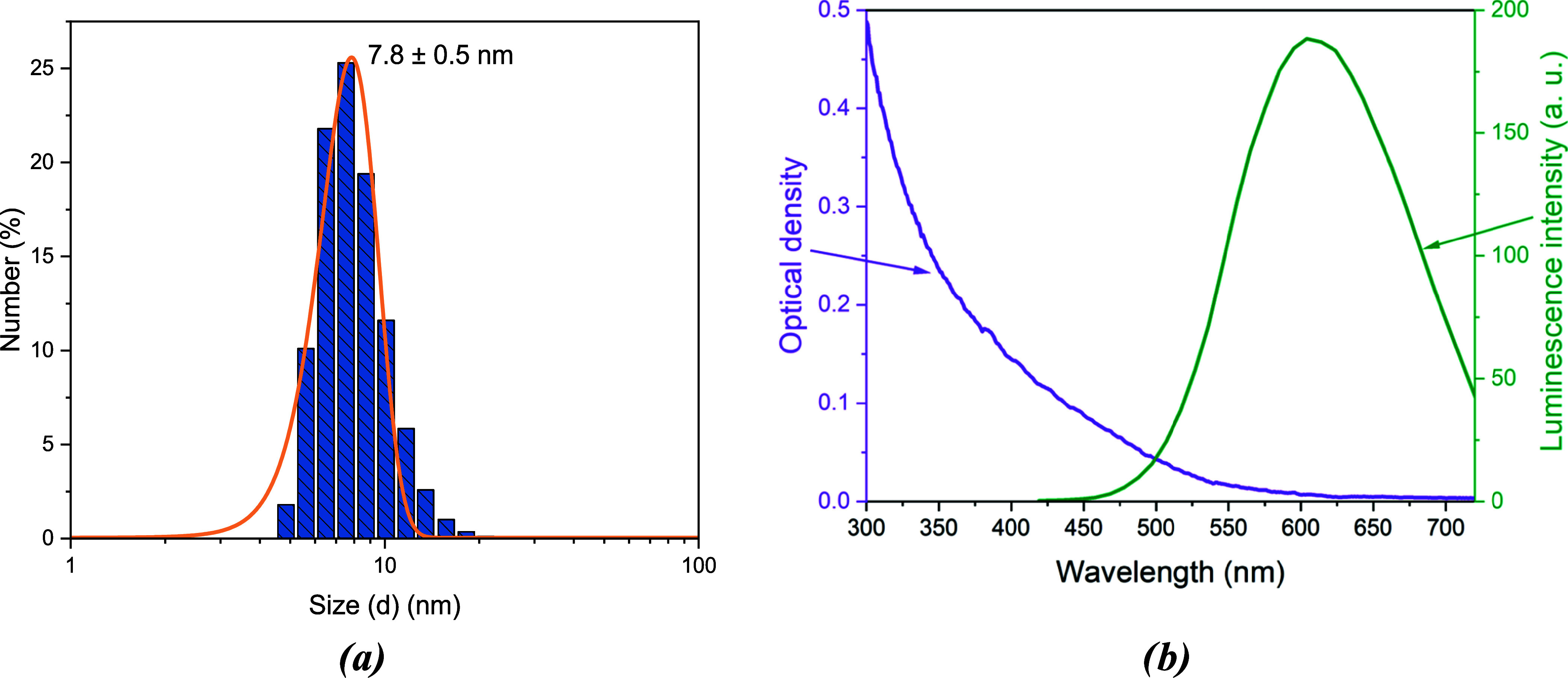
(a) Size distribution of AgInS2/ZnS core–shell quantum dots obtained with dynamic light scattering (DLS) analysis; the average size of quantum dots is 7.8 ± 0.5 nm. (b) Optical density (left axis) and luminescence (right axis) spectra of AIS QDs in water.

**Figure 3:**
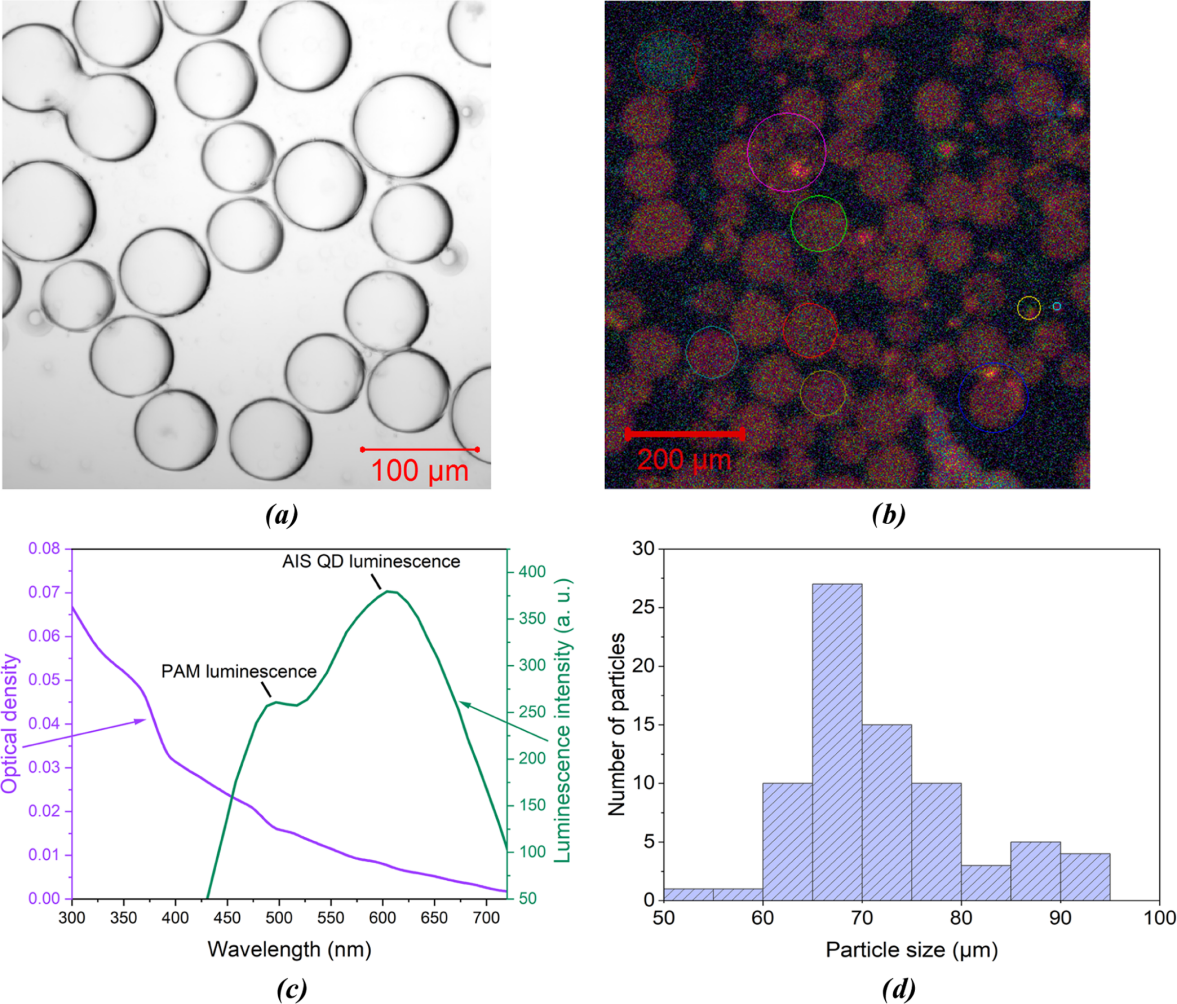
(a) Bright-field and (b) fluorescent images of microspheres doped with AIS QDs obtained with a confocal microscope. Circles represent areas from which luminescence signals were collected and averaged. (c) Optical density (left axis) and luminescence (right axis) spectra of microspheres with an AIS QD. (d) Microspheres: particle size distribution histogram.

An SF-56 spectrophotometer was used for optical density measurements (Figures 2 and 3). The luminescent properties of AIS QDs and polymer microspheres doped with AIS QDs were studied with a Zeiss LSM 710 laser scanning confocal microscope Zeiss LSM 710. A 405 nm CW laser (2 mW power) was used to excite the luminescence of quantum dots. The photoluminescence intensity was registered on the whole wavelength range at once. For this procedure, a 10 μL drop of AIS QD solution in water and a 15 μL drop of AIS QD-doped microspheres in oil were deposited on a glass slide. A 20×/0.75 objective was selected for the sample study.

### Characterization of Quantum Dots and Acrylamide Microspheres

The quantum dots obtained via hydrothermal synthesis were characterized via dynamic light scattering (DLS) analysis (Figure 2a) and zeta potential (ζ) determination. The formation of stable aqueous nanocolloids predominantly due to electrostatic stabilization (ζ > −30.2 mV) was confirmed. The histogram of nanoparticle size distributions clearly indicates the nanoparticle average size of 7.8 ± 0.5 nm. These values are in harmony with the data of 6.7 ± 0.8 nm obtained from scanning transmission electron microscopy (STEM), which demonstrated the spherical nature of the produced QDs.^24^

The absorption band of AIS QDs in water (Figure 2b) starts at 600 nm and extends into the UV range. The maximum of the photoluminescence intensity was registered at the wavelength of 625 nm for AIS QDs in solution.

We acquired a series of optical microscopy images of the microspheres with a scanning confocal microscope in order to estimate the variation of the polymeric particle size and its possible impact on the luminescence of QDs inside. The average diameter of a microsphere is 72 μm, with a minimum diameter of 55 μm and a maximum diameter of 95 μm (Figure 3d). Such a deviation is due to the different pressures used in the technique.

The luminescence decay was examined via luminescence kinetics by fluorescence lifetime imaging microscopy (FLIM). For this, a PicoQuant MicroTime 100 laser scanning luminescence microscope with the option of measuring the luminescence decay times was used to measure the time parameters of luminescence. This microscope is equipped with a pulsed laser with the same wavelength as was used in the confocal microscope (i.e., 405 nm). A variable pulse repetition rate (250 kHz for AIS QDs in solution and 2.5 MHz for AIS QDs in polymer microspheres) and a pulse duration of 70 ps were used. The difference in the pulse repetition rate is due to a significant difference in the decay times of AIS QDs in solution and AIS QDs in polymer microspheres since choosing the same repetition rate for both samples made it impossible to obtain correct decay curves.

## Results

### Optical Properties of AIS QD-Doped Microspheres

**Figure 4:**
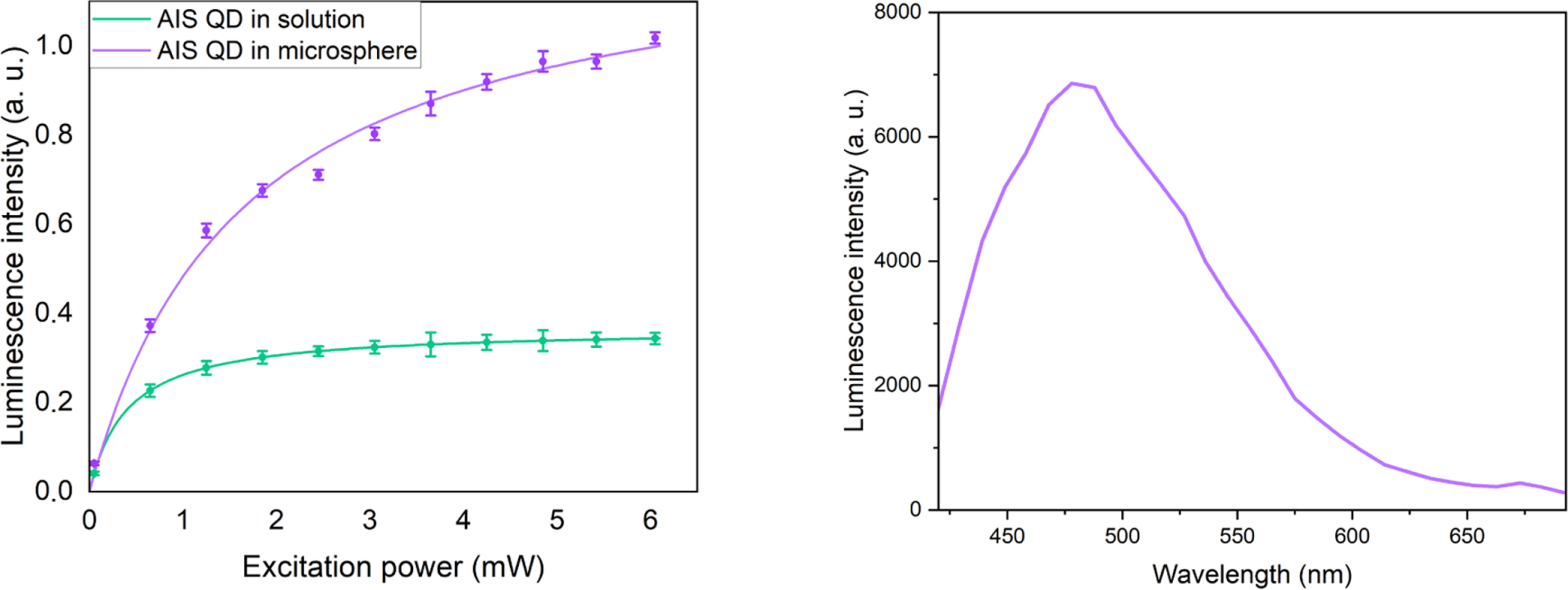
(a) Dependencies of the luminescence intensity of AIS QD solution and the single microsphere doped with the AIS QD on the excitation power. (b) Luminescence spectra of undoped polymer microspheres.

Figure 3 shows the bright-field and fluorescent images of the AIS QD-doped microsphere sample. The absorption band of microspheres with AIS QDs (Figure 3c) starts at 700 nm and extends into the UV range. The luminescence spectrum, measured with a confocal microscope at 405 nm excitation, is presented in Figure 3c (green curve). The maximum photoluminescence intensity was registered at a wavelength of 625 nm for microspheres with AIS QDs. A separate peak for microspheres is attributed to the luminescence from the polymer itself (see Figure 4b).

### Photoluminescence and Its Decay

In aqueous solutions, the AIS core–shell quantum dots have been reported to achieve photoluminescence quantum yields of 77% on average and up to 83% at best.^25,26^ When AIS QDs are incorporated into acrylamide microparticles, they have been shown to maintain their high luminescence, with the quantum yield reaching up to 58.27%.^26,27^ The matrix helps to stabilize the quantum dots and prevent aggregation, preserving their optical properties. As a step further, the influence of the excitation power on the luminescent properties of polymer microspheres doped with quantum dots was analyzed.

Using the same confocal microscope, we varied the excitation power from 0.05 to 6.05 mW with a 0.60 mW step for both samples: QD solutions and a single QD-doped microsphere. The laser spot area was 0.16 mm^2^. As a result, we observed a saturation dependence of luminescence for QDs in solution; it started at ca. 2 mW, while the luminescence intensity for QDs in microspheres was growing (Figure 4a). It is an interesting fact since, as demonstrated for CdSe/ZnS quantum dots, the linear dependence of the luminescence intensity on excitation was expected at low intensities of excitation.^28^ The most intriguing result is the anomalous behavior of QD luminescence: the saturation of QD luminescence in the aqueous solution (Figure 4a, green curve) and increasing QD luminescence when placed in a polymer (Figure 4a, purple curve). As was previously reported for CdSe/ZnS core–shell quantum dots, such a saturation is related to changes of the excited-state lifetime of QDs in different media;^29^ however, it can also be a signature of the Purcell effect.^30^

The luminescence intensity of microspheres with AIS QDs noticeably rises as a result of the increasing excitation power. Although the further increase of the luminescence intensity is observed, luminescence from the polymer itself (the emission maximum is 485 nm, see Figure 4b) was not considered as making a noticeable contribution to the total signal since the peak of QD luminescence was registered at the wavelength of 625 nm. The excitation power ceases to affect the luminescence intensity of AIS in water when it reaches 2 mW.

We consider that the microfluidic synthesis of microspheres dilutes AIS solutions, resulting in the dilution of quantum dots by 26 times in relation to the stock solution. In turn, it leads to a weaker intensity of luminescence of QDs embedded in the microspheres, as luminescent signals are expected to be related to the concentration of QDs.^31^ This fact can also be taken into account when explaining the saturation behavior as it may be caused by concentration quenching and fluorescence reabsorption possible in the stock solution.^31^

Increasing the excitation power did not destroy microspheres since no decrease in the luminescence intensity was observed. This suggests the good photostability of the AIS-doped polymer particles.

**Figure 5:**
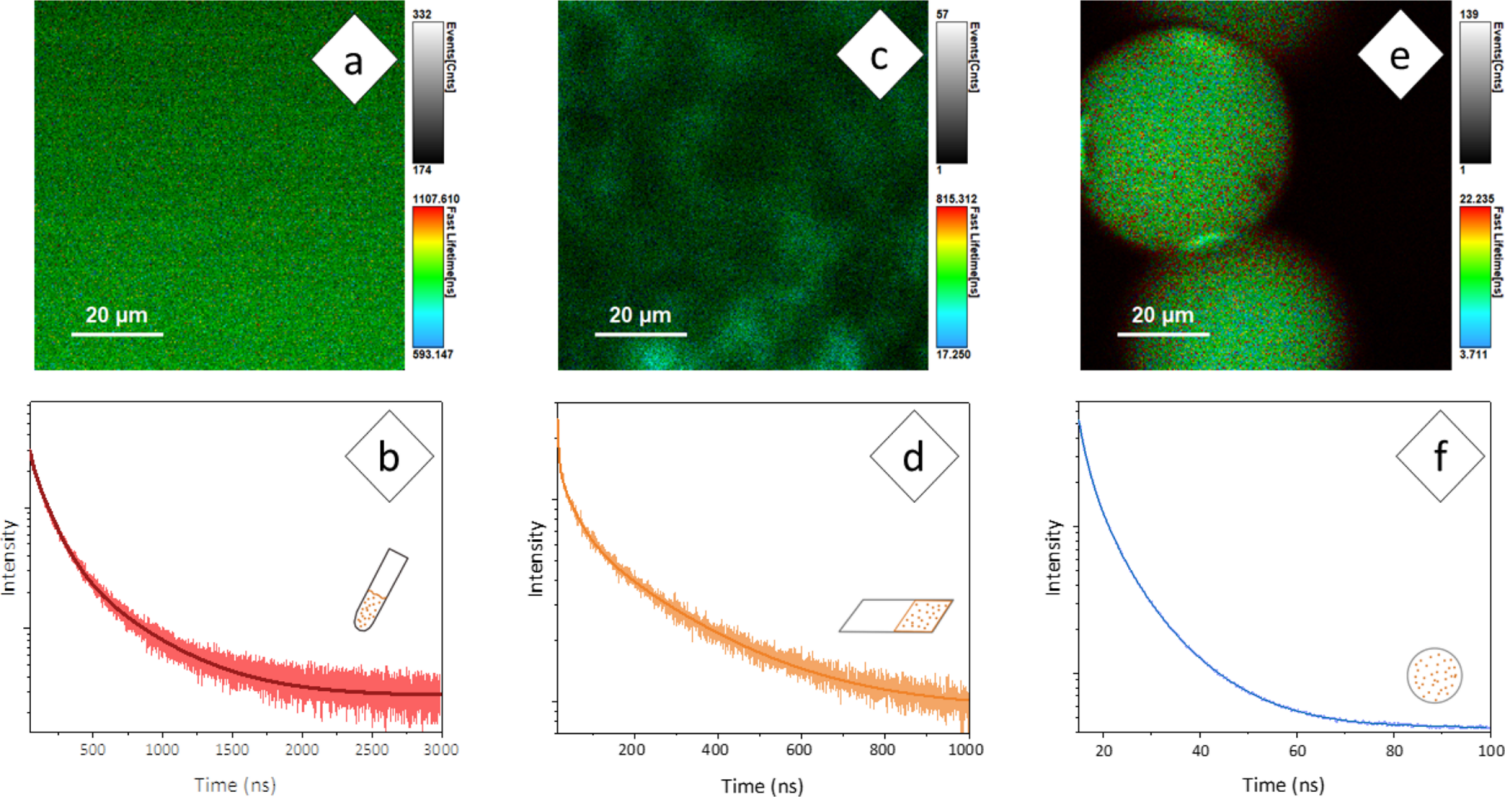
Fluorescence lifetime imaging microscopy (FLIM) images and luminescence decay curves with their approximations of (a, b) AIS QDs in water, (c, d) AIS QDs in the flat thin polymer film, and (e, f) AIS QDs in the microsphere; the color of the image corresponds to the decay time of the luminescence, and the brightness corresponds to the intensity.

The luminescence decay curves are shown in Figure 5. These figures represent FLIM images, decay kinetics, and their approximations for the QD stock solution in water (Figure 5a) and QDs embedded in the microspheres (Figure 5c). Additionally, for an assessment of the possible impacts of the polymer on QD luminescence, we prepared a thin film (by agglomeration of spheres) with similar QDs in the polymer (Figure 5b). The relaxation profile, *F*(*t*), is represented as a triple-termed exponential decay and could be fitted with the following equation:
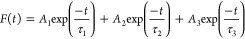
1where *A*_1_, *A*_2_, and *A*_3_ are weights of the corresponding exponential terms containing decay components τ_1_, τ_2_, and τ_3_, respectively. Results of measured decay times and weight coefficients of the corresponding components are summarized in Table 1. The average decay time, τ_AVG_, was estimated as
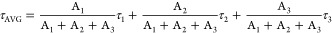
2It is clear that embedding QDs inside the polymer reduces their lifetime (Table 1). However, our FLIM analysis demonstrates that the lifetime for QDs in polymer microspheres is drastically reduced in comparison with the lifetimes of QDs both in water and in the layered polymer. Comparing the QDs’ lifetime in water and in the layered polymer, this difference reaches about 25% only, while QDs in microspheres show a shortening of their lifetime by 26 times in comparison with QDs in water and 19 times in comparison with the layered polymer. The FLIM technique also allowed to observe values of the so-called “fast lifetime”. The brightness encodes the intensity, while the color encodes the average fast lifetime corresponding to the average photon arrival time with respect to the preceding laser pulsed excitation. Though fast lifetimes are plotted on the basis of simple single-path calculation algorithms, they represent the lifetime component τ_3_ (this is the prevailing component, see Table 1) and its distribution in all samples. Their values are presented in the FLIM scale bars (Figure 5): 1107.6, 815.3, and 22.2 ns. According to their maximal values, the lifetime reduction for the fast component is 50-fold for the pair of QDs in water and QDs in microspheres and 37-fold for the pair of QDs in the thin film and QDs in microspheres.

**Table 1:** Decay Times and Amplitudes Used for the Approximation of Decay Kinetics

	τ_AVG_, ns	τ_1_, ns	τ_2_, ns	τ_3_, ns	*A*_1_, %	*A*_2_, %	*A*_3_, %
AIS QDs in the colloidal solution	91	440.0 ± 5.0	116.0 ± 2.0	18.0 ± 1.0	7.7	41	51.3
AIS QDs in the film	68	236.0 ± 5.0	39.0 ± 3.0	3.2 ± 1.0	23.7	26.9	49.4
AIS QDs in spheres	3.5	10.9 ± 0.1	4.2 ± 0.1	1.4 ± 0.1	11.8	35.4	52.8

## Discussion

To rationalize the saturation behavior of QD luminescence alongside drastic lifetime reduction, several mechanisms should be considered. The four most important mechanisms are detailed below.

### Passivation

This mechanism is directly associated with the quantum dots used.

The incorporation of quantum dots into a polymer matrix contributes to surface passivation and enhances the emission intensity. Recent studies have shown that passivation of the QD surface explains the reduction of lifetimes of QDs in water compared to in the polymer.^32^ For AgInS_2_/ZnS QDs, the luminescence lifetime is reduced by half in the polymer.

### Effect of the Medium, Collisional Quenching, and Aggregation

These effects, while having slightly different origins, often lead to luminescence quenching. Collisional quenching, a diffusion-mediated process, depends on the diffusion of QDs or quenchers in the medium, increasing nonradiative deactivation due to collisions. This affects the fluorescence lifetime without necessarily changing the absorption spectrum. High emitter concentrations can cause concentration quenching, a similar nonradiative relaxation process, i.e., collision and nonradiative relaxation of emitters, that is called concentration quenching.^33^ Similarly to concentration quenching, quenching may be caused by aggregation of quantum dots.^34^ Additionally, the medium’s characteristics, such as the polarity, temperature, and aggregate state may affect the kinetics of luminescence.^35,36^ All three processes give a good explanation for the lifetime changes that we observed when using different media and concentrations of QDs. However, they do not account for the difference between QDs in the layered polymer and in microspheres.

### Energy Transfer

The optical density spectrum of microspheres with AIS QDs is more complex than that of QDs in solution due to the polymer’s contribution, visible as an additional shoulder near 500 nm in the luminescence spectra (Figures 3c and 4b). Our investigation indicates that 405 nm laser radiation excites both QDs and the polymer when mixed, which is accompanied by a subsequent energy transfer from polymers to QDs. This explains why QD luminescence in the aqueous solution becomes saturated at intensities faster than those of QDs in polymers. Indeed, placing QDs in polymers affects the luminescent properties of QDs significantly^37^ since we observe the shortening of their lifetime in layered polymers (Table 1). Again, the lifetime for QDs in microspheres is drastically reduced in comparison with the lifetime of QDs in the layered polymer, which cannot be explained by energy transfer.

Thus, while the passivation, medium effects, and energy transfer contribute to the observed phenomena, they do not fully explain the drastic reduction in the QDs’ lifetime within polymer microspheres. This study underscores the complex interplay of these mechanisms and involving a new one.

### Purcell Effect

The enormous reduction of the luminescence lifetime when QDs are embedded in a microsphere cannot be merely associated with the acceleration of nonradiative decay, as when QDs were embedded in a polymer film of the same material, the reduction in the luminescence lifetime was insignificant. Under these conditions, it is natural to associate the reduction of the lifetime with a reduction of radiative decay times, which in the case of a microsphere is apparently caused by the Purcell effect and is absent in the case of a thin polymer film. In other words, the observed effect, the Purcell effect, is a significant growth of the QDs’ spontaneous emission rate when they are incorporated into a resonant cavity. Indeed, polymer microspheres may support whispering-gallery modes (WGMs) since their refractive index (1.502 [taken from refractiveindex.info, the original refs (38,39)]) is slightly higher that the refractive index of the medium (1.467 [taken from sigmaaldrich.com]); it is not considering QDs, whose refractive index is still unknown but expected to be about 2.5 since the similar compound has a refractive index > 2.55 at 625 nm.^40^ It is known that polymer WGM cavities have *Q*-factors up to 10^3^–10^6^.^41^ Using the formula for the Purcell factor^9^
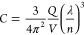
3one can estimate lifetime reduction as
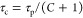
4where *Q* and *V* are the quality factor and the effective mode volume of the WGM, λ is the wavelength, *n* is the refractive index, τ_p_ is the lifetime of QDs in the film, and τ_c_ is the lifetime reduced due to the Purcell effect. For an assessment of the lifetime reduction, we may use the following approximations. We used the refractive index of the polymer as 1.5; the effective mode volume can be estimated using ref (42) as ≈800 μm^3^ for 72 μm-sized spheres. The *Q*-factor of a particular WGM is defined by surface and bulk absorption as well as scattering, radiative losses, contaminations, and even nonlinear absorption.^43^ As described in ref (43), radiative (curvature) losses vanish exponentially with increasing size, e.g., for a particle with a diameter 15 times larger than the wavelengths of the WGM, curvature losses can be omitted since the *Q*-factor is >10^11^. One of the prevailing factors affecting the total *Q*-factor budget for our case is the value of material losses caused by QD absorption. Following the formula for *Q*-factor contribution by material losses^43^
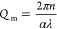
5where α is absorption, we can estimate the total *Q*-factor. In our case, absorption was calculated using the measured optical density value of QDs in microspheres (Figure 3c) at 625 nm (*D* = 0.05) and the Beer–Lambert law
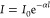
6where *l* is the optical path length, *I*_0_ is the initial intensity, and *I* is the intensity of light that traveled through the sample. The calculated absorption value was 0.12 cm^–1^; thus, the value of material loss contribution is *Q*_m_^–1^ ≈ 1.1 × 10^–6^. On the other hand, since many polymers are known as tending to form porous surfaces, it is just as important to evaluate losses due to the roughness of the surface, as described by the following expression:^43^
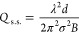
7where *d* is the microsphere diameter, σ is the root-mean-square (RMS) size, and *B* is the correlation length of surface inhomogeneities. The average microsphere diameter was 72 μm. Using the following values: σ = 0.3 nm and *B* = 3 nm, reported for glass surfaces,^44^ we obtain the *Q*_s.s._^–1^ value of 5 × 10^–9^. As a reference value for acrylamide, we may also use σ = 1.2 nm;^45^ however, even assuming that the RMS size of the microsphere surface is larger than the RMS size for glass by an order of magnitude, the losses due to surface inhomogeneities amount to 5 × 10^–8^, making the roughness contribution to be neglected. Thus, the *Q*-factor is approximately 10^6^. This value is in good agreement with values of *Q*-factors previously reported for polymer microresonators ∼10^6^,^41,46^ silica microspherical resonators coated with polymers ∼10^7^,^47^ and WGM resonators coated with quantum dots ∼10^7^.^48^

Using formula 3 for the Purcell factor, *C*, we assess it as equal to 7. This means that the lifetime reduction factor has to be 8. We admit that this is a very rough estimation since even for different modes in one microsphere, their Q-factor and effective mode volume may vary significantly; nonetheless, it shows an approximate correlation of the fluorescence lifetime reduction; by its order, it is in decent agreement with the experimentally observed averaged lifetime reduction as well as component τ_3_. Direct measurement of WGM resonances and their *Q*-factors are hardly possible since for whispering-gallery modes, we assessed their free spectral range (FSR) for AIS QD-doped microspheres using the following formula:^41^
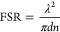
8The calculated FSR for two radial modes for the 72 μm resonator is of 1.2 nm. However, spherical WGM resonators of such diameters support an enormous number (at least several hundreds)^49^ of orbital and azimuthal modes and TE and TM modes as well; even though the Q-factor tends to 10^6^, the mode’s full width at half-maximum is expected to be at least 1 pm. Moreover, taking into account that QDs have their own size distribution (Figure 2a) that causes broadening of their fluorescence band, WGM modes are overlapped and separate resonances are unresolvable with our spectral devices.

## Conclusions

In this work, we introduce a microfluidic dripping technique for fabricating polymer microspheres doped with AgInS_2_/ZnS quantum dots. This method shows high productivity and potential for future enhancements to control the microsphere diameter and dopant concentration. The chosen AgInS_2_/ZnS quantum dots are water-soluble and having low toxicity, paired with a biodegradable polymer suitable for biosensing applications. Our results demonstrate that the quantum dots embedded in the microspheres exhibit a bright fluorescence and good photostability.

Additionally, we observed that the photoluminescence saturation by excitation of quantum dots in an aqueous solution was reduced when the same quantum dots were incorporated into polymer microspheres. Previous studies noted the intensity saturation, but our focus is on the luminescence decay. The average decay times measured were 91 ns for quantum dots in water, 68 ns for quantum dots in a polymer thin film, and 3.5 ns for quantum dots in polymer microspheres. This significant shortening is likely due to the Purcell effect with an assessed Purcell factor of 19, indicating a substantial lifetime reduction. We support our explanation with the potential occurrence of whispering-gallery modes within the microspheres. Future experiments should include measuring quantum yields of luminescence and comparing them with lifetime data as well as direct measurement of whispering-gallery modes using a high spectral resolution. This research also reveals the potential of AgInS_2_/ZnS quantum dots for nonlinear optical devices, offering a wide range of lifetime control.

In summary, our microfluidic dripping method provides an efficient approach to creating fluorescent nanocrystal-doped polymer microspheres with promising applications in biosensing and nonlinear optics. The observed Purcell effect and potential for whispering-gallery modes within these microspheres underscore the significance of our findings. Future work will further elucidate the quantum yields and the direct measurement of these modes, advancing the understanding and application of these quantum dot-doped microspheres in various fields.

## Notes

The photograph used for Figure 1 was taken by one of the coauthors, N.F. Similarly, the photo used for the TOC picture was captured by another coauthor, K.K.
